# American Academy of Dental Science

**Published:** 1893-10

**Authors:** 

**Affiliations:** American Academy of Dental Science


### Reports of Society Meetings.

### AMERICAN ACADEMY OF DENTAL SCIENCE.

THE regular monthly meeting of the American Academy of Dental Science was held in the Boston Medical Library Association rooms, May 3, 1893, at 7.30 P.M., President Brackett in the chair.

*President Brackett.*—We come to the subjects of discussion for the evening, which are as follows: 1. "What are the advantages and disadvantages of the use of the matrix? (*a*) With gold. (*b*) With plastics." 2. "Should examining boards have power to grant certificates of qualification to undergraduates?" I believe Dr. Ainsworth is particularly interested in the first subject, and we shall be grateful to him if he will open the discussion.

### DISCUSSION.

*Dr. Ainsworth.*—To me the advantages in the use of the matrix in compound approximal fillings of bicuspids and molars is very great. To begin with, let me say that I am, as a rule, an uncompromising advocate of contour work. I am also firmly of the opinion that we cannot better preserve approximal surfaces than by using non-cohesive or soft gold at the cervical wall. To make such a filling entirely of soft gold, either with or without a matrix, seems to me impossible. By the use of the matrix I fill approximal cavities one-third or two-thirds full with soft gold, building out a proper contour, and then finish with cohesive gold. It seems to me that it would be impossible to work without a matrix and attain the same positive result. And then, if the matrix is properly formed and adjusted in place, the finishing of the filling becomes a simple matter. I have never been able to use the matrix to advantage with gold fillings in the front teeth.

*Dr. Clapp.*—I have here some specimens that I prepared for an altogether different purpose, but as many of them require the use of the matrix, I make no apology for bringing them.

An advantage of the matrix for gold fillings is this: it often saves cutting away tooth-substance in the process of shaping a cavity. For instance, in the case of an approximal cavity in a devitalized bicuspid. The cavity may be quite large, and at the cervical margin may be U-shaped. To start a filling with gold in such a cavity without a matrix is rather difficult. To facilitate this process the tooth is oftentimes cut squarely at the cervical portion, thereby reducing the tooth at its weakest point. These cavities can be filled with a matrix without this sacrifice of tooth-structure. Dr. Ainsworth has spoken of other advantages, such as the more rapid introduction of the filling and the labor saved in the finishing process. I cannot conceive of a case where a properly-adjusted matrix could be a disadvantage.

*Dr. G. T. Baker.*—I have used the matrix with a great deal of satisfaction, and have made it of various materials, some of which I have brought for your inspection. Here is a sheet of copper silvered on one side; it was the first material I ever used, and was suggested by Dr. H. A. Baker eight or ten years ago. This one is thin steel; and here is a piece of German-silver, also very thin. I have never used aluminum, but have seen it somewhere suggested for this purpose. We have not yet been able to successfully solder aluminum, which stands to its disadvantage. The other materials spoken of are very easily manipulated and soldered. I believe in making a matrix for each individual case; it can be done in a very short time, and is more satisfactory than using those we buy ready made. Here is a strip of brass which I use to a certain extent, which gives me a great deal of satisfaction.

*Dr. Allen.*—I wish to bear testimony to the great value of the matrix in my work. I use it principally in plastic fillings of all kinds. I have had some unfavorable results from its use in filling cavities with gold, but I attribute the want of success more to my lack of familiarity with the method than to the method itself. In using the matrix with gold fillings I have sometimes found them a little soft at the periphery, as I have been unable to thoroughly condense the gold around the outer edge of the cavity at the beginning of the operation. To avoid this I first lay the foundation of the filling, covering the edges of the enamel before applying the matrix, after which I adjust it, and complete the operation.

*Dr. Ainsworth.*—I would like to ask Dr. Allen whether he commences his filling with cohesive or soft gold. I refer to compound fillings in bicuspids or molars.

*Dr. Allen.*—Soft gold.

*Dr. Ainsworth.*—Do you have difficulty in condensing it at the cervical wall?

*Dr. Allen.*—Not more than at any other point.

*Dr. Ainsworth.*—The very places where you speak of failure I feel very positive of. May I ask what you use for a matrix?

*Dr. Allen.*—The steel plate.

*Dr. Ainsworth.*—And conform it to the original contour of the tooth?

*Dr. Allen.*—As nearly as possible.

*Dr. Ainsworth.*—What is your method of holding it?

*Dr. Allen.*—I hold it by means of ligatures or by a wedge.

*Dr. Ainsworth.*—May it not be that you hold it too positively at the walls?

*Dr. Allen.*—Possibly.

*Dr. Ainsworth.*—I am using a very thin cold-rolled steel, a steel that has a temper very similar to piano-wire. You can bend it to any desired position, and yet it has something of a springy nature. I form it as nearly as possible to the original contour of the tooth, and hold it in position by various methods. I often use the Perry separator, and then insert a piece of orange-wood at the cervical wall, not too tightly, but just tight enough to bring the matrix towards the tooth. Beginning with soft gold, I can force the matrix away, not only at the cervical wall, but all the way up, so that when it is taken off I have a cavity which is uniformly too full. Two-thirds of the surface is made of cohesive gold. With proper burnishers I can burnish the filling very nearly to a finish, leaving only a trifle to be done by the disks and strips.

*Dr. Allen.*—I thank Dr. Ainsworth for his suggestions, and would like to ask him if he shapes the edge of the cavity in any peculiar manner?

*Dr. Ainsworth.*—I shape the cavity with reference to filling it with a matrix, not having it too rounding, but very nearly on a plane, with just the feather-edge taken off. I then pack two-thirds or three-quarters of the filling with hand-pressure, beginning with my cohesive foil when I have yet sufficient undercut to hold the filling. I do not know how extensively the matrix is used, but I feel that it is not adopted as it would be if all understood its advantages. I was for a time prejudiced against it, feeling that it would be impossible to pack gold properly in the places of which Dr. Allen speaks. And I thought that the finishing would consist more in working the tooth down to the filling than in working the filling down to the tooth, but this has not proved to be the case.

Another advantage in the matrix is that it enables us to restore the original shape of the tooth without previous separation. In the case of teeth which have never been filled or filed away, I do not care for separation obtained by the wearing of a wedge for two or three days or a week.

*Dr. Allen.*—My method of managing cavities where the matrix seems to be indicated is to use the adjoining tooth as a matrix.

*Dr. Ainsworth.*—You get a point by that means, but you do not get a uniform fulness. Your leverage is from only one spot. I venture to say that I can save one-fourth to one-third the time in filling an approximal cavity by using the matrix.

*Dr. Meriam.*—I think I am on record all I need to be as regards the materials to be used for matrices. I would speak again of the value of the optician's pliers, which give just the right curve for bending a matrix into position. Opticians use them for adjusting the spring of an eye-glass. They are not made in this country, but are imported by the American Optical Company.

One of the great values of matrices is in Dr. Clapp's method of confining a softer material so that it can be built on advantageously with a hard one. It thus helps out in those places where it would otherwise be difficult to retain a hard filling. I use it for this purpose quite extensively.

It is useful in molars in filling with amalgam when the cavity involves the mesial and distal surfaces. A matrix can encircle the whole tooth and be left on until the amalgam hardens.

There is an advantage in breaking up your amalgam chips and either mixing them with the fresh amalgam or embedding them in it. I think there is less contraction in an amalgam filling which is built into a hole. We speak of simple crown cavities as the simplest form of filling, and the question is suggested, "Do we not make a better filling when the filling-material is held on all sides against our pressure than when it is open on one side?" If we do, then it seems to me the matrix has a great value on that account alone. That idea is one which I do not think is on record. We know that of late years various drugs used in the form of pills or tablets which were formerly compounded by the assistance of the various gums are now made to hold together by a powerful pressure. In that way you get a condensed substance. Just in proportion as a substance is porous, just in that proportion will it be acted on chemically. A phosphate filling made with a matrix is a harder filling, and, consequently, less liable to solution than one made without such pressure.

I generally hold my matrix in place with a steel wedge, of which I have had several made. They are wedge-pointed, and just fit into my automatic plugger. After driving them in, a slight turn leaves them in position. I also use the ordinary pine wedge, which has, to a certain extent, the advantage which Dr. Ainsworth speaks of in the orange-woods,—that is, it is not too rigid. I whittle the pine out to a proper size and then compress it with a pair of pliers. After putting in the wedge a drop of water causes it to swell and hold the matrix firmly in place, though not so firmly that it will not give a little. I have some of this soft pine with me, which I bought for horticultural purposes, and shall be pleased if the gentlemen will take as much as they wish of it.

*Dr. Brown.*—Ever since I have been in practice I have used the matrix wherever I could, thanks to Dr. Ainsworth's instructions. I find it helps me very much indeed, and I am sure I have accomplished results which I could not have without it.

*Dr. Clapp.*—I find it absolutely impossible to get along without the matrix in combination fillings of amalgam and gold. One of the great advantages of the matrix is the help it gives in making contour fillings. I have felt conscience-stricken repeatedly on account of the time used in filling when I have heard men who are called rapid operators give their experience. I had a short time ago a young man whose teeth had been cut down unmercifully, so that the spaces between most of them were V-shaped. At the cervical portion the teeth were close together, and I think in every instance where they had been filled decay had reappeared under the fillings. After I had worked some time on this young man, he told me, without any request on my part, who had had charge of his teeth, and I am sorry to say that the gentleman who did the work I consider to be the very best rapid operator there is in New England. Now, I have to contour those teeth in order to get them in any decent condition. The gums are very much inflamed from the impacting of food in the wedge-shape spaces. If it was not for the use of the matrix my work would certainly be very much more uncomfortable for the patient than it now is.

In teeth badly broken down by caries, the matrix is of great advantage. This specimen is a good representative. It is decayed down to the alveolar margin, not only on the distal surface, but a little on each side,—that is, both towards the palatal and labial surfaces. In such cases I adjust the matrix first and then put the rubber dam over it.

*President Brackett.*—I think we should express our sense of appreciation for this group of practical specimens that Dr. Clapp has been so kind as to put before us.

*Dr. Meriam.*—Having a distal cavity in a second molar, with the wisdom-tooth partly erupted, I made a matrix with an upper lip, which was bent backward, and formed a support for the rubber dam, which, without such help, could not be adjusted. This form of matrix changed a difficult operation into an easy one. You do not know how much cold-rolled steel can be forged without drawing the temper until you have tried it. You will find that you can make a knuckle by making a dent in the steel at the point desired.

*Dr. Stevens.*—There is one class of cavities that has not been mentioned in connection with the use of the matrix, and that is those shallow approximal cavities where the teeth are sometimes so sensitive that we cannot get undercuts to retain the filling. In such cases I oftentimes apply the matrix and fill in against it with amalgam, and then put soft cement between the amalgam and the wall of the cavity to insert the amalgam, and then finish with either gold or amalgam. In that way I get a filling that will stay without undercuts, and the work is very easily done.

*Dr. Baker.*—I sometimes make a matrix from a Parmly Brown polishing strip. The strip is bent about the tooth with pliers, and the ends soldered with soft solder. I used to solder with eighteen-carat gold solder, but the soft solder is just as good, besides being inexpensive.

*Dr. Stevens.*—I do not understand how you can solder a matrix and then get it over the contour of a molar tooth.

*Dr. Meriam.*—In the case I referred to the molar-tooth was so badly decayed there was not much contour.

*Dr. Ainsworth.*—I have had several cases lately of bicuspids that were very badly decayed, both upon the mesial and distal surfaces, so much so, in fact, that it was impossible to get sufficient retention to hold any filling excepting a cement. The pulps were alive, and enough of the buccal portion of the tooth remained to make a good appearance. In these cases I used a matrix made by passing a piece of German-silver entirely around the tooth, drawing it together with the pliers, removing, and soft soldering. With a burnisher I arranged the contour on either side, having previously separated the teeth. By this means I have been able to fill a large part of the cavities on either side with soft foil. By building the top of the filling with cohesive gold, the distal filling held the mesial, and *vice versa*. And the result was a finely-shaped tooth, and one of great durability. This could not have been done without the aid of the matrix.

*President Brackett.*—Unless some one wishes to speak further on the subject we will consider the discussion closed on this matter and proceed to the second topic, which is, "Should examining boards have power to grant certificates of qualification to undergraduates?"

*Dr. Stevens.*—No.

*Dr. Meriam.*—I should like to ask if that applies to examining boards generally, or to examining boards in Massachusetts? It is no use for us to express our wishes in matters over which we are not likely to get any control. The same power which gives the colleges the right to grant degrees or to establish qualifications for graduation also appoints the examining boards. Now, the question is, how far our examining boards have a right to refuse to examine any man who chooses to present himself. We must not hold the idea that an examining board created by Massachusetts could refuse to examine any person, or, having examined a person, could refuse to issue a certificate if the examinations were satisfactorily passed, whether the person be an undergraduate or a graduate.

*Dr. Stevens.*—I would say, in reply to Dr. Meriam, that the topic reads, "*Should* examining boards have the power to grant certificates?" It does not say, "*Have* examining boards the power?" That is another question. It seems to me that the time has passed when examining boards should have the power to issue certificates to undergraduates. We have men come up before college professors who do not creditably pass the examination, and they go before the dental board, and are allowed to practise.

*Dr. Allen.*—Dr. Stevens's remarks suggest a matter that came under my direct observation. About a year ago a student of one of our dental colleges, nineteen years of age, after having had a little less than nine months' actual experience in dentistry, went before the State Board of Examiners and applied for a license to practise dentistry. The result was that in the course of two or three weeks a letter came to him, addressed in my care, which read something like this: "Dear Doctor,—We have the pleasure of informing you that you have successfully passed the examination of the Massachusetts State Board of Registration in Dentistry, and that your certificate will be sent you in a fow days." He received the certificate in due time, and with something less than ten months' experience came out a full-fledged dentist.

*Dr. Meriam.*—I do not see why the examination of the Board of Examiners should be so low. Some students pass who have not even matriculated.

*Dr. Brown.*—I graduated from the Boston Dental College in 1890, and the examination of the State Board was a very rigid one. Had I not taken the full course at the college I feel sure that I would not have been competent to pass. I do not understand how undergraduates are able to pass, though I know of a number who have done so. They must have been let off easily by the examiners.

*Dr. Clapp.*—I would like to ask Dr. Brown if, in his estimation, the examination which he received from our State Board was sufficient and satisfactory from a professional view.

*Dr. Brown.*—My examination was.

*Dr. Clapp.*—Do you think that the examination you passed would be sufficient to qualify any person to practise?

*Dr. Brown.*—It was certainly severe, both theoretically and practically.

*Dr. Allen.*—There must be a great many exceptions to the rigid examination which Dr. Brown passed. This young man that I speak of is an intelligent young man, but not remarkable as a student. He was not a graduate at the time of taking the State examination, and has not graduated yet; and it is doubtful if he will succeed in passing the dental school examinations this coming year. In the first year examinations he failed on anatomy, chemistry, and another study; he was conditioned on these three studies when he applied for examination at the State Board. His first filling was put in only six months or so before his State examination, and they passed him and gave him his certificate.

*Dr. Payne.*—I graduated in 1889, and took my State examination at the same time, and was only asked six simple questions.

William H. Potter, D.M.D.,
*Editor American Academy of Dental Science.*

